# Awareness of hypertension and factors associated with uncontrolled hypertension in Sudanese adults

**DOI:** 10.5830/CVJA-2013-035

**Published:** 2013-08

**Authors:** Fawzi A Babiker, Elkhalifa Lamia A, Mohamed E Moukhyer

**Affiliations:** Department of Physiology, Faculty of Medicine, Kuwait University, Kuwait; Department of Community Medicine, Faculty of Medicine, Ahfad University for Women, Sudan; Department of Community Medicine, Faculty of Medicine, Ahfad University for Women, Sudan

**Keywords:** hypertension, blood pressure, awareness, risk factors, gender differences, stress, family history

## Abstract

**Background:**

The incidence of hypertension (HTN) has increased rapidly in the Sudan in the last few years. The aim of this study was to determine the prevalence of uncontrolled HTN and the risk factors associated with it in Sudanese adults.

**Methods:**

This study was cross sectional. Data were collected using structured questionnaires filled in during interviews with subjects visiting referral clinics in Khartoum, the capital city of Sudan. Blood pressure (BP) was measured using a digital sphygmomanometer. A digital balance was used for determination of body weight and a traditional cloth tape measure was used for measuring height, for calculation of body mass index.

**Results:**

This study included 200 subjects, 46% male and 54% female. In the whole study, 82% of subjects (*p* < 0.001) were on hypertension drug treatment. Of these, 64% had their BP controlled to normal standards set by the World Health Organistion (< 140/90 mmHg). The prevalence of uncontrolled BP was significantly (*p* < 0.001) higher in males (61%) compared to females (15%). When the risk factors of HTN were considered, 54% of the subjects had a positive family history of HTN and 52% were smokers. Uncontrolled BP was found to be significantly (*p* < 0.001) higher in smoking males (43%) compared to females (4%). It was also high in people with higher education (55%) and workers (41%). In these groups, when genders were considered separately, uncontrolled hypertension was significantly (*p* < 0.01) higher in males than females with higher education (67 and 40%, respectively), and in workers (86 and 10%, respectively). Uncontrolled HTN was associated with overweight and obesity in 45 and 29% of the subjects, respectively. Most of the interviewed subjects were not aware of the consequences of HTN and its associated risk factors.

**Conclusions:**

Uncontrolled HTN was associated with risk factors of HTN and lifestyle, and was more prominent in the male gender. The ignorance of the interviewed subjects about HTN, its associated risk factors, changes in lifestyle and adherence to taking the medication may have been a major factor in the prevalence of uncontrolled HTN.

## Abstract

Hypertension (HTN) is universally accepted as one of the most important risk factors in the development of cardiovascular disease (CVD), stroke and renal disease.^1^-^3^ There has been a considerable increase in the prevalence of HTN in the Middle East during the last few years. In some Arab countries HTN has become a major health problem.^4^ This drastic increase in incidence of HTN is specifically caused by a combination of many parameters, including family history,^5^ change in lifestyle, dietary habits and environmental factors.^6^

Sudan is considered one of the leading countries in Africa for the prevalence of HTN.^7^ However, a proper national registry on HTN is not available in Sudan and evaluation studies are rarely done.^8^ Nevertheless, a recent study showed an inreasing incidence of HTN in Sudan,^9^ which may result in serious health problems in the near future if no appropriate measures are taken.

The prevalence of HTN may be a result of the marked shift in the Sudanese diet, which has resulted in increased overweight and obesity.^4^ Some studies have claimed the prevalence of HTN is a cause of the tremendous increase in renal insufficiency.^3^

Increased awareness, follow up and control of HTN in industrialised countries has resulted in a decreased tendency to morbidity and mortality from cardiovascular disease.^10^ To reach the level of improvement attained in developed countries, epidemiological studies on the risk factors, control methods, control levels, lifestyle, adherence to medication, and awareness will be crucial for setting control strategies in Sudan.

In this study we investigated the prevalence of uncontrolled HTN in Sudanese adults and determined the factors that may be responsible for the poor control in the study participants. We also acquired essential information on risk factors and their effects on outcome of the control of HTN.

## Methods

This study was a clinic-based, cross-sectional study. The study population was a group of 200 hypertensive patients selected randomly from referral clinics in the teaching hospitals (Omdurman, Khartoum North and Khartoum). Subjects underwent interviews using a standard questionnaire, collecting data on personal information, presence of concomitant diseases such as diabetes and renal failure, family medical history, medication use, salt intake, and awareness of HTN and its control.

All the interviewed subjects were considered for the study except those who were diagnosed with kidney failure before the start of our study. These subjects were excluded because their HTN may have been secondary hypertension or influenced by the kidney failure and the subjects’ body fluid volumes. All subjects invited to participate in the study responded positively and participated, except three (two because of lack of time and one declined participation for no reason).

At the beginning of the study, blood pressure (BP) of the subjects was measured using digital sphygmomanometers (Omron, MX3 Plus, Kyoto, Japan), which had been validated to the European Society of Hypertension’s international protocol.^11^ BP measurements were done on one occasion and repeated three times with five-minute intervals between them. The subjects were in a seated position. The averages of these measurements were used for further analysis. HTN was systolic blood pressure (SBP) above 140 mmHg and/or diastolic blood pressure (DBP) above 90 mmHg (WHO, Guidelines for the Management of Hypertension),^12^ or use of antihypertensive medication by the study subjects.

Body weight was measured using a digital balance and height was measured using a traditional cloth tape measure. Body mass index (BMI) was calculated from the weight and height using the formula, BMI = weight (kg)/height (m^2^). Overweight was defined as BMI = 25.0–29.9 kg/m^2^.

All subjects interviewed in this study were adults. All personal information and measurements were kept confidential. Authorisation was obtained from the ethics committee at Ahfad University for Women, Omdurman, Sudan before the start of the study and an informed consent was signed by the selected individuals before filling in the questionnaire.

## Statistical analysis

All statistical analyses were done using the statistical package SPSS (version 17.0, SPSS Inc, USA). Results were summarised as percentage for all variables. Chi-squared (χ^2^) test was used for the analysis of factors associated with HTN. The data were analysed using the Student’s *t*-test to determine means for the variables; *p* < 0.05 was considered statistically significant.

## Results

The study population comprised 200 hypertensive patients selected randomly from those who were visiting referral clinics in Khartoum. All study subjects were adults, 20 years of age and older. The subjects interviewed were 92 (46%) males and 108 (54%) females. Of the study subjects, 148 (74%) were married, 44 (22%) were single, four (2%) were widows and four (2%) were divorced.

The education level of the subjects varied widely; 32 (16%) were illiterate, 44 (22%) had primary school level, 80 (40%) had secondary education and 44 (22%) higher education (university and graduate). The employment pattern was 68 (34%) unskilled manual workers, 88 (44%) government employees and 44 (22%) were self-employed or in the private business sector.

The majority of subjects (144, 72%, *p* < 0.001) were only hypertensive with no other non-communicable diseases such as stroke or diabetes. HTN in combination with diabetes was found in 48 (24%) patients and HTN, combined with stroke in eight (4%). There were 112 (56%) subjects with a family history of hypertension, 84 (42%) with diabetes, and four (2%) with other diseases among their direct blood relatives.

In this study HTN was found in 92 (46%) of the subjects during a routine general check-up. It was recognised in the other 108 (54%) after the start of complications. Although 56 (28%) of the subjects did not remember their BP readings during their last visit to the clinic, BP monitoring in the clinics showed controlled BP (120–140/60–90 mmHg) (self-referred) in 76 (38%) subjects, and 68 (34%) had uncontrolled BP (> 140/> 90 mmHg) (Table 1).

**Table 1:** Data Representing, Health Status, Family History, How HTN Was Detected For The First Time, Historical Measurement Of Blood Pressure In The Clinics And BP Measurement During Our Study (*n* = 200)

	*Variables*	*n*	*%*
Health status	Hypertension	144	72
	Hypertension + diabetes	48	24
	Hypertension + stroke	8	4
Family history	Hypertension	112	56
	Diabetes	84	42
	Other	4	2
HTN detection	Routine check-up	92	46
	After complaint	108	54
BP monitoring	120–140/60–90	76	38
(mmHg)	>140/>90	68	34
	Do not know	56	28
BP measured in study	120–140/60–90	128	64
(mmHg)	>140/>90	72	36

In our BP check-ups during this study, 128 (64%) subjects showed controlled and 72 (36%) uncontrolled BP and only 2% showed very high BB levels (Table 1). The uncontrolled BP was significantly (*p* < 0.001) higher in males (56, 61%) than females (16, 15%) when genders were analysed separately (Table 2). Marital status had no influence on the lack of BP control when the genders were pooled, however, a high prevalence of uncontrolled HTN was shown in 48 males (62%) compared to eight females (11%, *p* < 0.001) when they were considered separately (Table 2).

**Table 2:** Comparison Of Gender, Marital Status, Educational Level And Employment And Their Relationship With Level Of Control Of HTN In Subjects With Controlled Or Uncontrolled Hypertension (*n* = 200)

			*Blood pressure*				*Uncontrolled hypertension*			
			*Normal*		*High*		*Male*		*Female*	
*Variables*	*n*	*%*	*n*	*%*	*n*	*%*	*n*	*%*	*n*	*%*
Gender										
Male	92	46	36	39	56	61	–	–	–	–
Female	108	54	92	85	16	15	–	–	–	–
Marital status										
Single	44	22	28	64	16	36	8	50	8	29
Married	148	74	92	62	56	38	48	62	8	11
Widows	4	2	4	100	0	0	0	0	0	0
Divorced	4	2	4	100	0	0	0	0	0	0
Education										
Illiterate	32	16	24	75	8	25	8	50	0	0
Primary school	44	22	44	100	0	0	0	0	0	0
Secondary school	80	40	40	50	40	50	32	67	8	53
Higher education	44	22	20	45	25	55	16	67	8	40
Employment										
Workers	68	34	40	59	28	41	24	86	4	10
Government-employed	88	44	52	59	36	41	28	50	8	25
Self-employed	44	22	36	82	8	18	4	50	4	11

Although uncontrolled BP was found in eight (25%) of the illiterate subjects, surprisingly, the lack of control increased with increasing educational level, as it was found in 40 (50%, *p* < 0.01) subjects with secondary education and in 24 (55%, *p* < 0.01) of the higher educated subjects when both genders were considered together. The highest prevalence was found in males compared to females (32, 67% and eight, 25%, respectively) (*p* < 0.01) with secondary education, and 16 (67%) and eight (40%), respectively (*p* < 0.03) with higher education (Table 2) when genders were considered separately.

Uncontrolled HTN was found in 28 workers (41%), in 36 (41%) government employees and in eight (18%) self-employed subjects. However, a high prevalence of uncontrolled HTN was found in 24 (86%) (*p* < 0.01), 28 (50%) (*p* < 0.01) and four (50%) males compared to four (10%), eight (25%) and four (11%) females among workers, government employees and selfemployed subjects, respectively, when genders were considered separately (Table 2).

In this study we also investigated awareness among the study subjects, their willingness and effort to control their BP, and their motivation to change their lifestyle. For the variable following up on their BP and monitoring it at home, 160 (80%) (*p* < 0.001) of the subjects did not monitor their BP at home, and 184 (82%) patients took their medication as prescribed by the doctors. Of all subjects studied, 172 (86%) (*p* < 0.001) showed a desire to normalise their BP. To follow up on their PB, 132 (66%) subjects visited doctors on a regular basis. Surprisingly, for control of BP and change of lifestyle, 104 (52%) subjects did not decrease their salt intake in their diet, and 120 (60%) still ate Faseekh, which contains large amounts of salt (Table 3).

**Table 3:** Data Representing The Desire Of Patients To Contribute To Decreasing Their Bp By Monitoring It At Home, Adhering To Drug Use, Control Visits To The Doctor And Dietary Changes (*n* = 200)

*Variables*		n	%
BP monitoring at home	Yes	40	20
	No	160	80
Drug intake as prescribed	Yes	164	82
	No	36	18
Efforts to control BP	Yes	172	86
	No	28	14
Control visits to doctor	Yes	132	66
	No	68	34
Salt in daily meals	Eat with family	104	52
	Eat low salt	96	48
Eating salty food (extra salt)	Yes	120	60
	No	80	40

**Fig. 1.**
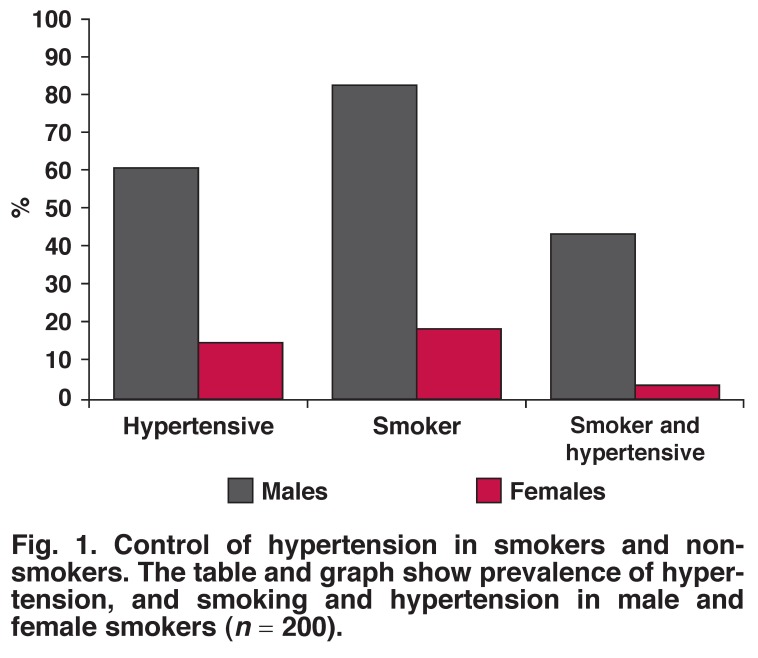
Control of hypertension in smokers and non-smokers. The table and graph show prevalence of hypertension, and smoking and hypertension in male and female smokers (*n* = 200).

In the whole study group, the number of non-smokers was 104 (52%) compared to 96 (48%) smokers. Of these, smoking was found in 76 (83%) males and 21 (19%) females (*p* < 0.001). The number of subjects who were hypertensive and smokers was 40 (43%) males and four (4%) females (*p* < 0.001) (Fig. 1).

**Fig. 2.**
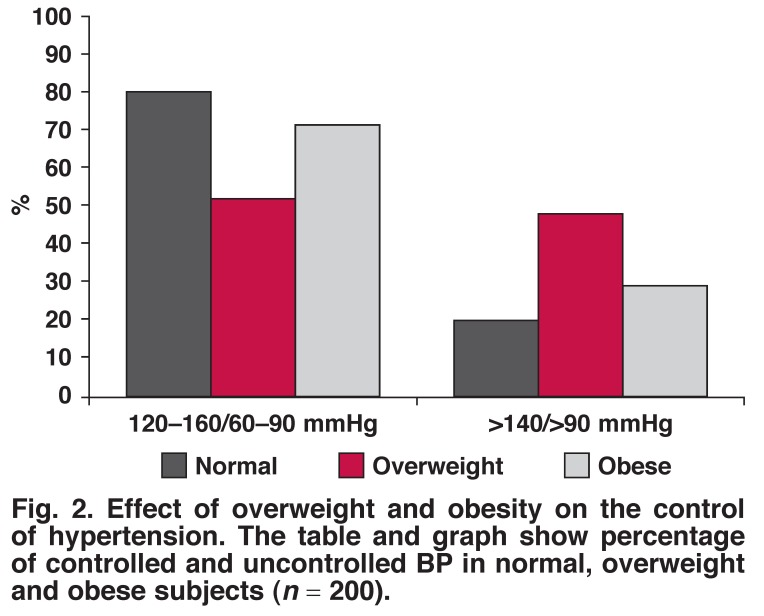
Effect of overweight and obesity on the control of hypertension. The table and graph show percentage of controlled and uncontrolled BP in normal, overweight and obese subjects (*n* = 200).

BMI estimation showed that 20 (10%) of the study subjects were of normal weight, 84 (42%) were overweight and 96 (48%) were obese (Fig. 2). In this study, the relationship of HTN control with overweight and obesity showed that a higher BMI had a more detrimental effect on HTN control than a normal BMI. With regard to BP control, there were 44 (52%) (*p* < 0.03) subjects in the overweight and 60 (71%) in the obese category compared to16 (80%) normal-weight subjects. Surprisingly, BP control in the overweight category was less compared to obese subjects (Fig. 2).

## Discussion

In this study, uncontrolled BP was found in 34% of the subjects, which is in line with studies in other countries where high levels of uncontrolled BP were recognised.^13^,^14^ However, the percentages of uncontrolled BP in this study were higher than that obtained in other studies.^15^,^16^ This tendency to have uncontrolled BP is common, even in developed countries such as the USA.^17^,^18^ Our study suggests a similar prevalence of uncontrolled HTN in Sudan compared to other countries. However, there has been a increase in incidence of uncontrolled HTN in Sudan over the last few years. Further studies are required to determine suitable methods of BP control to decrease the percentage of uncontrolled hypertension in the population.

When we considered the risk factors of HTN, this study revealed that HTN control was better in females than males. These results are similar to those from previous studies where females were better protected and achieved better BP control than males.^19^,^20^ The prevalence of uncontrolled HTN was also found to be higher in males than females, who were proven to be protected by the female gender due to the presence of the hormone oestrogen.^21^ Furthermore, male subjects are less likely to seek medical care.^22^

Surprisingly, the level of education showed a positive association with uncontrolled hypertension in our male subjects, as the presence of uncontrolled HTN increased with the educational level. These results are in line with a previous study done in Sudan.^23^ There is no explanation for this association but we can speculate that HTN in these subjects may have been complicated by other factors such as stress at work and a sedentary lifestyle.

Both marital status and employment were found in this study to be associated with uncontrolled BP in males, although there was no positive relationship between marriage and lack of control of HTN in all studies done to date.^24^ The negative impact of marriage on HTN control in our study may have been due to complications from other stresses, risk factors and lifestyle changes. Low income in Sudan may also be an important stress factor in marriage. Stress at work could be due to work demands and low salaries, which would influence the outcome of BP control.^10^,^25^

The female subjects in all educational groups, marital and work categories showed better BP control than the males. This may have been because of the presence of oestrogen, which is protective against HTN.^21^ Moreover females were known to be better than males in adherence to medication.^26^

Other risk factors such as positive family history for HTN were also evident in this study. This is not surprising as Shehata *et al.*^5^ found relatives of hypertensive subjects to be more likely to have HTN early in life.^5^ The risk of occurrence of HTN was found to be greater in subjects with hypertensive first-order family members.^27^

When we evaluated the awareness of HTN and its control, our data showed a very poor appreciation of HTN and its associated risks in the study population. The study showed that 54% of our subjects discovered their HTN when the complications of the disease became apparent. In the rest of our subjects, HTN was detected by chance in a routine check-up. These finding suggest a poor healthcare system and health education programmes. These results confirm the lack of a national data registry on HTN in Sudan, which points to a low level of screening and follow up.^28^ Lack of awareness is very significant in the prevalence of HTN and uncontrolled high BP.^10^,^16^ More attention must be given to HTN as it is a common risk factor for stroke^29^ and kidney disease,^30^ which are recognised to be rapidly increasing in Sudan.

Lack of awareness was evident in our studied subjects. Compliance with drug usage as prescribed by the doctor was poor in our study subjects; 18% did not take their medication as prescribed. In other studies, 11% lack of compliance with drug use was considered a serious cause of poor treatment of HTN.^31^ Some researchers stated compliance with medication to be of vital importance for good results in the control of HTN.^18^,^32^,^33^

Lack of awareness in our study subjects can be viewed in several ways. Only 20% of the study participants went for checkups or monitored their BP at home, 14% did not change their habits to achieve suitable pressure levels and 34% did not visit doctors on a regular basis. Green et al. found that follow up with doctors and monitoring BP at home resulted in better control.^34^ Control of HTN was also influenced by the high sodium intake found in our study subjects. Increased sodium intake and a high-salt diet has been proven to be an important aspect in the prevalence of HTN in the Sudanese population.^35^-^37^

In this study, uncontrolled HTN was found to be prevalent in smoking males compared to smoking females. In many other studies, smoking has been recognised as a risk factor for HTN.^38^,^39^ BP levels were found to be higher in hypertensive smokers than in hypertensive non-smokers.^40^ Gender differences with regard to smoking have not been examined before, but our data are in line with other studies,^40^,^41^ where smoking was shown to interfere negatively with the control of HTN.

Overweight and obesity were common among our study subjects. From this it is evident that overweight and obesity are among the important risk factors of HTN in the Sudanese population. Obesity was also noted by Elmahdi *et al*.^42^ in a Sudanese population. A large number of the subjects in our study who had uncontrolled HTN were overweight or obese. High BMI is proven to be an important risk factor for HTN.^43^,^44^ Indeed our finding supports the notion that persistent overweight and obesity can interfere with the efficacy of hypertension drugs.^45^,^46^

## Conclusion

Uncontrolled HTN was associated with lifestyle and risk factors for HTN and was more prominent in the male gender. The lack of awareness in the subjects about HTN, its associated risk factors, changes in lifestyle and adherence to the medication may be a major factor in the prevalence of uncontrolled HTN in Sudan.
